# Expanded Hemodialysis Enhancement in Middle Molecule Clearance for Patients With Low Blood Flow Rates of Tunneled Dialysis Catheters

**DOI:** 10.7759/cureus.64323

**Published:** 2024-07-11

**Authors:** Lorenzo Aterini, Stefano Aterini, Barbara Vadalà, Francesco Ravaglia, Fiamma Balboni, Marco Gallo

**Affiliations:** 1 Nephrology, School of Human Health Sciences, Azienda Ospedaliera Universitaria (AOU) Meyer Children Hospital, University of Florence, Florence, ITA; 2 Hemodialysis Centre, Istituto Fiorentino di Cura e Assistenza (IFCA), Florence, ITA; 3 Laboratory Service, Istituto Fiorentino di Cura e Assistenza (IFCA), Florence, ITA

**Keywords:** hemodialysis, large middle molecules, uremic toxins, medium cut-off membrane, expanded hemodialysis, blood flow

## Abstract

Introduction: Expanded hemodialysis (HDx), being based on medium cut-off (MCO) membranes, improves the removal of medium molecule uremic toxins. HDx efficacy has been proven with blood flow rates (Qb) of 350-400 ml/min, while low Qb have only been assessed in single sessions. We evaluated the effectiveness of HDx in patients with tunneled central venous catheters (CVCs) and low Qb over six months, comparing it with high-flux hemodialysis (HF-HD).

Methods: The study included 10 patients with a mean age of 79±12 years and mean Qb of 237 ± 12 ml/min. Reduction ratios (RRs) and predialysis serum levels were measured for β2-microglobulin (B2M), free κ and λ light chains (FLC), prolactin (PRL), interleukin-6 (IL-6), albumin, and urea after HF-HD and at one, three, and six months of HDx. Erythropoiesis-stimulating agent (ESA) resistance index (ERI) was also evaluated.

Results: B2M, κ-FLC, λ-FLC, and PRL RRs were significantly higher with HDx. IL-6, albumin, and urea RRs did not show a statistical difference between the two treatments. Predialysis B2M concentrations were significantly lower after three and six months of HDx, matching up to increased B2M clearance (spKt/V). A decrease in albumin concentrations was observed, with median levels significantly reduced at months seven and eight (35.3 and 35.5 g/L, respectively) but recovering afterwards. ERI was significantly lower during HDx, reaching a 30% reduction at month six.

Conclusions: HDx was feasible, safe, and superior to HF-HD in patients with low Qb rates of tunneled dialysis catheters. The present data expand options for HDx prescription, with particular regard for patients who cannot achieve high convective volumes due to inadequate vascular access.

## Introduction

Low molecular and hydraulic permeability hemodialysis membranes (low flux) generally provide high clearance of small solutes such as urea and creatinine but lead to inadequate clearance for medium molecules. High molecular and hydraulic permeability hemodialysis membranes (high flux) significantly increase the removal of larger molecules, reducing their cumulation, which is associated with complications such as amyloidosis, inflammatory activity, and endothelial dysfunction. These complications contribute to reduced quality of life, morbidity, and excessive mortality in hemodialysis patients. Recently, a new type of membrane has been developed, medium cut-off (MCO) membranes, which can improve the removal of medium molecules up to 50 kD, such as β2-microglobulin (B2M) and free κ and λ light chains (FLC), while demonstrating reduced permeability to albumin. Even if hemodialysis with this type of dialyzer is technically diffusion-based, most of the removal of large solutes occurs by convection through the so-called internal filtration mechanism, obtaining solute clearances approaching those of online post-dilution hemodiafiltration (HDF) without delivering convective replacement fluid [1-3].

MCO membranes form the basis of a new diffusion-based therapy called “expanded hemodialysis” (HDx) [4]. As well as the choice of hemodialysis membranes (low flux or high flux) and treatment modalities (diffusive or convective), blood flow rate (Qb) plays a key role that most influences the performance of hemodialysis modalities. High Qb increases the dose of diffusive and convective dialysis, and in convective methods, a relationship between survival and volume of reinfusion fluid has been observed [5-9]. Vascular access is the critical determinant for obtaining adequate Qb. Even if the native arteriovenous fistula still represents the gold standard, tunneled central venous catheters (CVCs) have become increasingly widespread as vascular access for hemodialysis, sometimes as the first vascular access. However, CVCs may present reduced hemodynamic effectiveness by not allowing adequate Qb.

In studies validating the efficacy of HDx treatment published so far, Qb of the order of 350-400 ml/min have been used [10-13]. The safety and efficacy of HDx treatment with low Qb (250 ml/min) have been evaluated only in single sessions [14,15]. Hemodialysis patients with low Qb can experience the possibility of not being able to reach an adequate dialysis dose, facing the risk of underdialysis. The aim of this study was to evaluate HDx treatment in patients who had tunneled CVCs as a vascular access, with Qb lower than or at the best of 250 ml/min, assessing middle-sized uremic toxin removal, during a six-month time, in comparison with traditional high-flux hemodialysis (HF-HD). Their clinical characteristics make them hardly recruitable in randomized trials, but they are not so uncommon in everyday hemodialysis practices. Biological markers of medium molecules, such as B2M, κ-FLC and λ-FLC, prolactin, and interleukin-6 (IL-6), were measured, alongside albumin, considering its possible loss during HDx treatment. Furthermore, the possible higher removal of medium molecules provided by HDx was investigated in relation to erythropoiesis-stimulating agent (ESA) dose prescription, measuring the ESA resistance index (ERI).

## Materials and methods

The prospective, single-center study included 10 anuric patients, whose clinical characteristics are reported in Table 1.

**Table 1:** Patients’ clinical characteristics and dialytic parameters.

Variables	Mean±SD or N
Age (years)	79±12
Dialysis vintage (months)	83±55
BMI (kg/m^2^)	23±4
Female/male	3/7
Comorbid diseases at study time	
Diabetes	5
Hypertension	5
Cardiovascular	3
Neoplasia	2
Chronic liver disease	1
Treatment duration (min)	239±6
Blood flow rate (ml/min)	237±12
Dialysis fluid flow rate (ml/min)	500

Three were female and seven were male, with a mean age of 79 ± 12 years (range 55-94 years), stable on a thrice-weekly hemodialysis program. The mean hemodialysis vintage was 83 ± 55 months (range 24-192 months). All of them had a tunneled catheter as a vascular access due to vascular issues with multiple failed attempts at establishing fistulas/grafts (seven cases), limited life expectancy due to active neoplasia (one case), severe cardiac disease and/or atherovascular disease with lower limb amputation (one case), and venipuncture refusal (one case). Half of them suffered from chronic disability, while the others were functionally independent. Underlying renal diseases were chronic glomerulonephritis (one patient), diabetic nephropathy (two patients), hereditary kidney disease (one patient), nephroangiosclerosis (four patients), and undiagnosed nephropathy (two patients). The anticoagulant used during dialysis was low-molecular-weight heparin (enoxaparin). Ultrafiltration was prescribed based on patients’ clinical needs. All included patients provided informed consent. The study was approved by the Scientific and Ethics Committee of the Istituto Fiorentino di Cura e Assistenza (Nr. 1/24/CTS).

Each patient underwent routine HF-HD treatment with high-flux Revaclear 500™ (Poracton, Baxter). Subsequently, they were started on MCO Theranova 500™ (Polyarylethersulfone, Baxter). All hemodialysis sessions were carried out with Artis Baxter hemodialysis machine, maintaining their usual parameters in both the hemodialysis treatments, HF-HD and HDx: mean dialysis duration 239±6 min (range 230-250 min), mean blood flow rate 237±12 ml/min (range 220-250 ml/min), and dialysate flow rate 500 ml/min. Blood samples for analyses were collected from each patient in the first dialysis session of the week at the end of HF-HD period and after one, three, and six months of HDx treatment.

Reported laboratory measurements included B2M, κ-FLC and λ-FLC, prolactin, IL-6, albumin, and urea. Their final concentration were corrected for the degree of hemoconcentration according to Bergström and Wehle [16]: postdialysis concentration correction = postdialysis concentration/{1 + ((predialysis weight - postdialysis weight)/(0.2 × dry weight))}. Reduction ratios (RR) were as follows: {(predialysis concentration - corrected postdialysis concentration)/predialysis concentration} x 100. Kt/V B2M was calculated according to Casino et al. [17]: spKt/V = 6.12 ΔW/W_post_ {1−log (C_pre_/C_post_)/log (1 + 6.12 ΔW/W_post_)}, where ΔW stands for net weight loss, W for weight, C for B2M concentrations, and pre and post for pre- and postdialysis values. Moreover, common electrolytes, uric acid, C-reactive protein (CRP), parathyroid hormone (PTH), transferrin saturation index (TSI), and hematological values were documented. Serum routine parameters were measured locally. κ-FLC and λ-FLC were measured using Freelite® (The Binding Site, Birmingham, UK), based on polyclonal-specific antibodies. IL-6 was measured by chemiluminescence immunoassay (Roche). Friedman two-way analysis of variance (ANOVA) for several related samples was used for comparison between groups. Wilcoxon signed-rank test was used for comparison between two related samples. Statistical analysis was performed using SPSS Statistics for Windows, Student Version 6.0 (Released 1993; SPSS Inc., Chicago, IL, USA).

## Results

As far as medium molecules were concerned, B2M RR with HDx treatments was significantly higher than that obtained with HF-HD treatment (Table 2).

**Table 2:** RRs after HF-HD (T0) and after one (T1), three (T3), and six (T6) months of HDx. In the significance level column, the first line shows the comparison between groups; the lines below show the comparison between paired groups. RR: reduction ratio; IQ: interquartile; B2m: beta2-microglobulin; FLC: free light chains; PRL: prolactin; IL-6: interleukin-6; HF-HD: high-flux hemodialysis; HDx: expanded hemodialysis.

	Median RR (%) (IQ range)				Significance level
	T0	T1	T3	T6	
B2m	69 (65-74)	70 (65-72)	74* (68-78)	74** (70-77)	p= 0.0172 *p= 0.0249 vs T0 **p= 0.0166 vs T0
κ-FLC	25 (23-31)	63* (57-69)	58* (48-64)	61* (55-69)	p=0.0003 *p=0.0051 vs T0
λ-FLC	25 (23-31)	43* (41-45)	44** (35-49)	42^§^ (31-47)	p=0.0129 *p=0.0117 vs T0 **p=0.0051 vs T0 ^§^p=0.0052 vs T0
PRL	47 (44-54)	61* (58-64)	64* (56-69)	59** (52-69)	p=0.0022 *p=0.0051 vs T0 **p=0.0144 vs T0
IL-6	21 (19-32)	40* (21-48)	23 (8-45)	25 (9-35)	p=0.1943 *p=0.0382 vs T0
Albumin	10 (6-18)	10 (6-17)	14 (8-19)	10 (4-19)	p=0.2561
Urea	77 (68-78)	73 (69-77)	74 (68-79)	74 (70-78)	p=0.8037

Since the first month of HDx treatment, κ-FLC and λ-FLC RRs were significantly higher than those measured in HF-HD (Table 2). λ-FLC RRs were lower than κ-FLC RRs due to their different molecular weights (45 kDa and 23 kDa, respectively). Prolactin RR during HDx was significantly higher than what was observed with HF-HD (Table 2). IL-6 RR slightly increased following the first month of HDx, but this difference did not attain statistical significance between groups at the different time sequences (Table 2). With regard to small molecules, no statistically significant difference was observed in urea RR between HF-HD and HDx (Table 2). Similarly, there was no significant difference in albumin RR at all the measured time intervals (Table 2). Patients were hemodynamically stable throughout the study period.

Apart from B2M and albumin, predialysis hematological parameters did not significantly change throughout the study (Table 3).

**Table 3:** Predialysis serum levels after HF-HD (T0) and after one (T1), three (T3) and six (T6) months of HDx. In the significance level column, the first line shows the comparison between groups; the lines below show the comparison between paired groups. B2m: beta2-microglobulin; IQ: interquartile; FLC: free light chains; PRL: prolactin; IL-6: interleukin-6; CRP: C-reactive protein; PTH: parathyroid hormone; TSI: transferrin saturation index; HF-HD: high-flux hemodialysis; HDx: expanded hemodialysis.

Variables (reference values)	Median predialysis laboratory data (IQ range)				Significance level
	T0	T1	T3	T6	
B2m (0.8-2.4 mg/L)	29 (27-33)	31 (29-33)	27* (24-31)	25** (21-29)	p=0.0009 *p= 0.0406 vs T0 **p=0.0284 vs T0
κ-FLC (3.3-19.4 mg/L)	134 (121-142)	135 (121-152)	130 (111-151)	141 (117-155)	p=0.5459
λ-FLC (5.7-26.3 mg/L)	110 (69-134)	121 (79-138)	126 (84-134)	115 (96-136)	p=0.5222
PRL (4.79-23.3 µg/L)	28 (19-63)	27 (20-62)	29 (16-60)	43 (22-57)	p=0.1544
IL-6 (≤7 pg/ml)	7.6 (5.0-10.1)	6.9 (4.0-13.7)	7.7 (3.3-17.3)	6.7 (5.8-9.8)	p=0.8471
Albumin (35-52 g/L)	38 (37-40)	36 (35-40)	39 (37-40)	36 (35-38)	p=0.1102
Urea (10-50 mg/dl)	156 (115-223)	148 (135-186)	135 (107-182)	160 (136-176)	p=0.6481
Bicarbonate (22-28 mmol/L)	22 (20-26	21 (20-22)	22 (20-24)	22 (20-26)	p=0.7747
Na (136-146 mEq/L)	139 (136-142)	139 (138-141)	137 (135-139)	137 (134-139)	p=0.2177
K (3.5-5.1 mEq/L)	6.0 (5.4-6.5)	5.6 (5.3-6.0)	5.8 (5.3-6.2)	5.9 (4.2-6.2)	p=0.3825
Ca (8.8-10.6 mg/dl)	9.0 (8.6-9.2)	8.9 (8.6-9.2)	9.3 (8.7-9.5)	9.2 (8.7-9.7)	p=0.2261
P (2.5-4.5 mg/dl)	6.3 (4.4-7.4)	5.3 (4.7-6.2)	5.1 (3.4-5.6)	4.3 (3.7-7.4)	p=0.1357
Mg (1.8-2.6 mg/dl)	2.40 (1.95-2.52)	2.20 (2.10-2.22)	2.35 (2.15-2.50)	2.05 (1.78-2.32)	p=0.1097
Uric acid (3.5-7.2 mg/dl)	6.1 (5.5-8.2)	6.3 (5.7-8.9)	6.0 (5.6-8.0)	5.8 (5.3-7.6)	p=0.0528
CRP (<5 mg/L)	4.7 (1.7-8.7)	4.9 (1.9-9.3)	4.6 (1.8-8.0)	4.4 (2.2-15.3)	p=0.3590
PTH (15-88 ng/L)	225 (129-338)	239 (148-315)	201 (136-350)	181 (135-370)	p=0.5751
TSI (30-50 %)	26 (20-31)	24 (18-30)	22 (19-30)	30 (25-43)	p=0.0762

**Figure 1:**
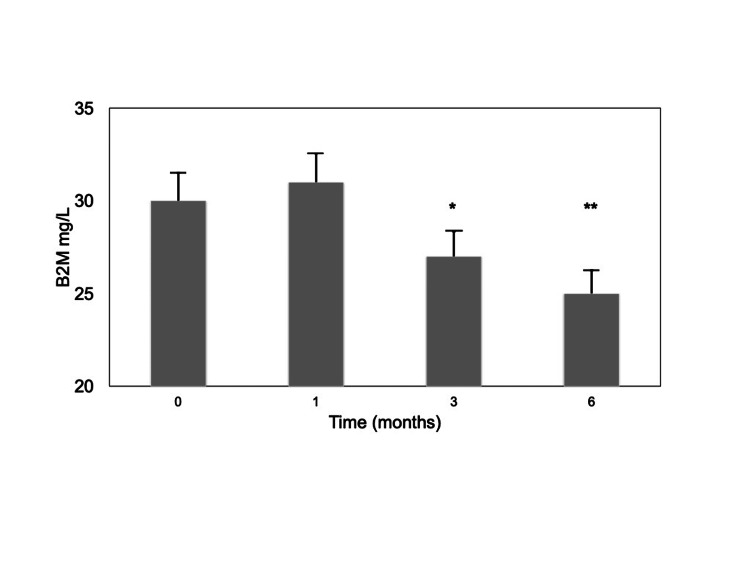
B2M predialysis serum concentrations after HF-HD (T0) and after one (T1), three (T3), and six (T6) months of HDx. Results are expressed as mean±SD. B2M: β2-microglobulin; HF-HD: high-flux hemodialysis; HDx: expanded hemodialysis. *p=0.040 vs T0; **p=0.028 vs T0.

Predialysis B2M concentrations were significantly lower after three and six months of HDx therapy (Table 3; Figure 1).

**Figure 2:**
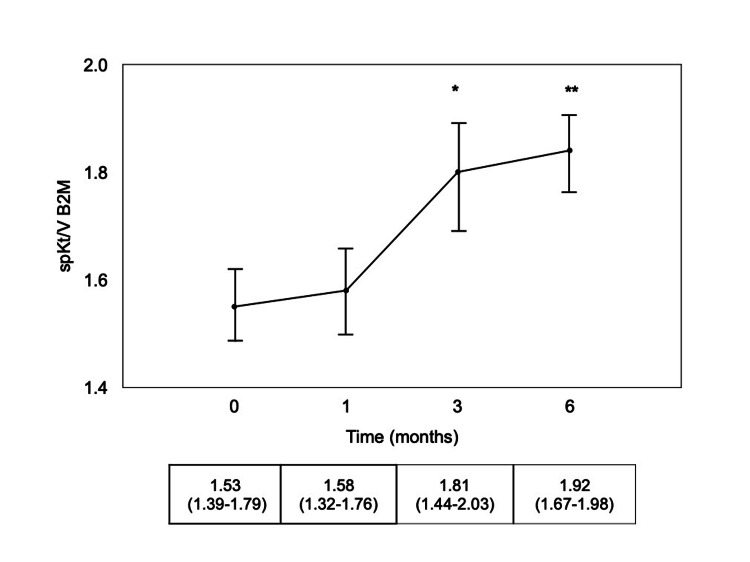
spKt/V B2M after HF-HD (T0) and after one (T1), three (T3) and six (T6) months of HDx. Results are expressed as mean±SE. In the row below, results are presented as median (interquartile range). B2M: β2-microglobulin; HF-HD: high-flux hemodialysis; HDx: expanded hemodialysis. *p=0.028 vs T0; **p=0.007 vs T0.

These results matched up to spKt/V B2M, which reached higher values at the same time intervals (Figure 2).

**Figure 3:**
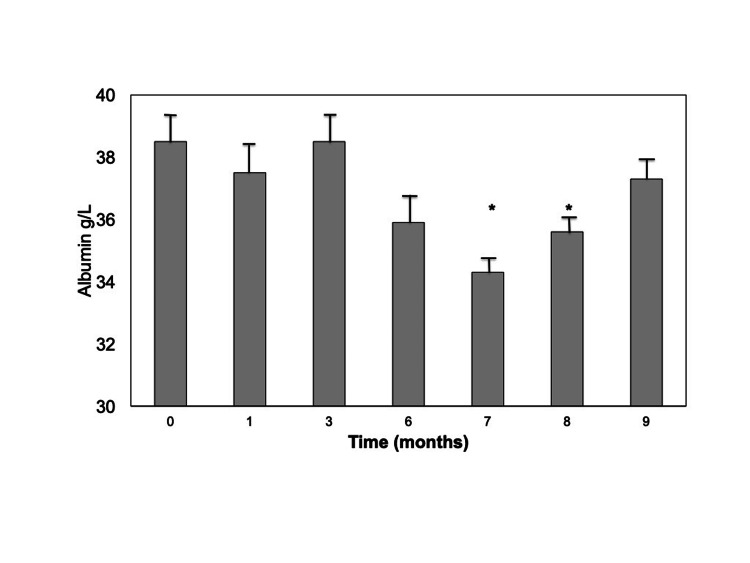
Albumin predialysis serum levels throughout the study period. Results are expressed as mean±SE. *p=0.0464 vs T0.

Changes in serum albumin levels were not statistically different, even though a decrease in albumin concentration was observed after six months of HDx treatment. In order to monitor potential changes in albumin serum levels, we extended albumin measurements for another three months, maintaining treatment parameters. Figure 3 shows that at months seven and eight, median albumin concentrations were significantly reduced (35.3 and 35.5 g/L, respectively), but they recovered afterwards (37.5 g/L).

**Figure 4:**
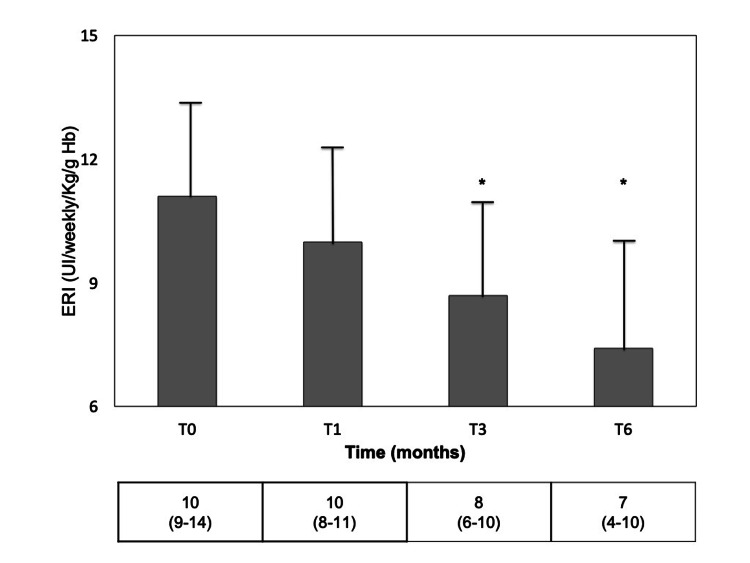
ERI after HF-HD (T0) and after one (T1), three (T3), and six (T6) months of HDx. Results are expressed as mean±SD. In the row below, results are presented as median (interquartile range). ERI: ESA resistance index; HF-HD: high-flux hemodialysis; HDx: expanded hemodialysis. *p=0.0077 vs T0.

In order to investigate ESA resistance, the ESA resistance index (ERI), defined as the weekly ESA dose per kilogram of body weight divided by Hb level (grams per deciliter), was used [18]. Patients received epoetin zeta throughout the study period. ERI was significantly reduced during HDx treatment at months three and six, reaching a 30% reduction at month six, keeping the iron prescription unchanged (Figure 4).

## Discussion

Hemodialysis patients with low Qb can experience the possibility of not being able to reach an adequate dialysis dose, facing the risk of underdialysis. This study showed that HDx treatment was suitable in patients with low Qb, improving the removal of medium molecule uremic toxins over 24 weeks of treatment in comparison with HF-HD. The patients included in this single-center study had a mean age of 79 years, older than the majority of patients reported in a meta-analysis comparing these two hemodialysis modalities with long enough follow-up time [19]. Their clinical characteristics and comorbidities make them not so uncommon in the real world of everyday hemodialysis practices, but they are underrepresented in clinical trials. Notwithstanding, a significant B2M RR of 74% was attained after three and six months of HDx treatment. These values are consistent with data recorded with studies performed with much higher blood flow rates [10-12,20]. In line with increased RRs, lower B2M serum levels were measured, which were mirrored by a sustained increase in its spKt/V.

Our observations are supported by a recent meta-analysis, which concluded that medium cut-off dialysis membranes not only lowered predialysis B2M in the short-term but also over long-term (≥12 weeks) treatment [19]. Albumin loss is of concern when MCO membranes are used in hemodialysis treatment since hypoalbuminemia is associated with a poor prognosis in hemodialysis patients. In our series, serum albumin showed a trend toward reduction, which reached the nadir at seven and eight months of HDx, followed by a return to normal, may be due to compensatory increased hepatic synthesis. Greater albumin removal with HDx than with HF-HD has been described, leading to decreased serum albumin concentration, but extending the duration of follow-up, the difference in predialysis serum albumin between HDx and HF-HD gradually decreased [19,21]. Similarly, Weiner et al. [12] also reported that in the first two months of the study the HDx group had slightly lower predialysis serum albumin than the HF-HD group. After 24 weeks, however, predialysis serum albumin levels did not differ significantly between the two groups. In comparison, in our series, the reduction in serum albumin was slower and later, which may be related to differences in blood flow rates between the two studies.

In spite of significant RRs since the first month of HDx treatment, PRL, κ-FLC, and λ-FLC predialysis concentrations did not change remarkably throughout the six months of study, similarly to other reports [22,23]. On the other hand, other studies described a decline in predialysis levels of κ- and λ-FLC [12,19]. Different findings could be explained by a higher tissue generation, suggesting that HDx treatment may not exert adequate influence on future predialysis levels, regardless of how efficiently it increases clearance. Among the other biochemical variables regularly measured in hemodialysis patients, phosphate only showed a lowering trend, which did not reach significance because of fluctuation in phosphate serum levels related to phosphate rich food assumption and/or inadequate compliance with prescribed phosphate binding drugs (sevelamer carbonate 5.2±2.0 g/day). Enhanced phosphate removal by HDx has been reported [20], but eating habits and therapies might influence results.

The negative correlation between end-stage kidney disease and chronic inflammatory status is suggested by elevated levels of IL-1, IL-6, tumor necrosis factor (TNF)-α, and CRP. We found that HDx had little to no effect on IL-6 removal or predialysis levels as well as on CRP concentrations, while HDx has been reported to reduce IL-6 with unchanged TNF-α levels [24]. On the other hand, no difference in serum levels of IL-6 with the reduction of TNF-α concentrations has been described under the same treatment [19,23]. Different results probably depend not only on the molecular weight, larger IL-6 size, and distribution volume but also on the inflammatory response and comorbid conditions of any single patient. Moreover, in uremic patients, chronic inflammation has been closely associated with ESA resistance. We observed a linear decrease in ERI over time throughout treatment with HDx. Similar results have been reported in other studies and meta-analysis [25-27].

A central role in erythropoietin resistance has been associated with erythropoiesis inhibition driven by cytokines and hepcidin, a peptide produced by inflammatory cells and involved in the anemia of inflammatory chronic disease [18]. The greater improvement in ESA resistance achieved by HDx than HF-HD has been associated with a larger removal of inflammatory cytokines, potentially influencing the iron metabolism in a hepcidin-independent manner [25]. In this study, observed changes in transferrin saturation index (TSI) and IL-6 levels did not reach statistical significance. Considering the previously reported non-uniform results for cytokine removal by HDx and the role of hepcidin in the development of ERI in the dialytic population, further studies are warranted to better define the mechanisms that determine ERI improvement with MCO membranes. The present data expand options for HDx prescription, with particular regard to patients who cannot achieve higher convective volumes in HDF because of inadequate blood flow rate, considering that it can be performed with common hemodialysis technology without requiring high volumes of replacement ultrapure fluid, an issue of the utmost relevance to the green dialysis concept [28]. More definite clinical end-points and impact on comorbidities and survival in hemodialysis patients will be derived from ongoing clinical trials [29].

## Conclusions

We aimed at assessing the feasibility and safety of HDx treatment in patients with vascular access difficulties, bringing about low Qb and the risk of underdialysis, not reaching an adequate dialysis dose. The small number of patients in a single-center study represents a limitation. However, in our setting, at the same low blood flow rates, MCO polyarylethersulfone membrane was superior to HF-HD for the clearance of middle-sized uremic toxins. HDx decreased predialysis concentrations of B2M only, without normalizing serum concentrations. Lower serum albumin levels were transient and followed by recovery. Finally, HDx reduced ESA resistance, suggesting that enhanced clearance of middle molecule hepcidin and inflammatory cytokines could be involved, as reported with convective therapies.
